# The effect of perceived organizational support on safety compliance in Libyan public hospitals: a cross-sectional study of the mediating role of proactive personality

**DOI:** 10.3389/fpsyg.2026.1877693

**Published:** 2026-09-29

**Authors:** Maryam G. Saaid, Ayşen Berberoğlu

**Affiliations:** 1 Institute of Graduate Research and Studies, Institute of Social Sciences, Business Administration Concentrated on Occupational Health and Safety, University of Mediterranean Karpasia, TRNC, Mersin, Türkiye; 2 College of Medical Technology Ajdabiya, Ajdabiya, Libya; 3 Department of Business Administration, Institute of Social Sciences, University of Mediterranean Karpasia, Mersin, Türkiye

**Keywords:** healthcare workers, hospital-based cross-sectional study, Libya, organizational support theory, perceived organizational support, proactive personality, safety compliance behavior

## Abstract

**Background:**

Perceived organizational support (POS) reflects employees’ beliefs that their organization values their contributions and cares about their well-being. Although POS has been linked to desirable workplace outcomes, less is known about the psychological processes through which it is associated with safety compliance behavior among healthcare employees, particularly in under-examined developing-country contexts.

**Objective:**

This study aims to evaluate proactive personality as an individual-level mechanism underlying the association between POS and safety compliance behavior in healthcare personnel.

**Methods:**

A hospital-based cross-sectional survey was conducted among 309 healthcare employees working in public hospitals in Ajdabiya, Libya. Data were analyzed using partial least squares structural equation modeling (PLS-SEM) with SmartPLS 4. Reliability and validity of the measurement model were assessed before testing the direct and indirect relationships using bootstrapping with 5,000 resamples (bias-corrected and accelerated, BCa).

**Results:**

POS was positively associated with proactive personality (*β* = 0.242, *p* < 0.001) and safety compliance behavior (*β* = 0.217, *p* < 0.001). Proactive personality was also positively associated with safety compliance behavior (*β* = 0.554, *p* < 0.001), the strongest path in the model. Proactive personality was also associated with a significant indirect association between POS and safety compliance (*β* = 0.134, *p* < 0.001), consistent with complementary partial mediation. The structural model accounted for 41.2% of the variance in safety compliance behavior and 5.9% of the variance in proactive personality.

**Conclusion:**

The findings indicate that proactive personality is associated with a stronger direct relationship with safety compliance behavior than organizational support is among healthcare workers in this sample. The study extends organizational support theory within the healthcare context by showing how individual differences relate to safety-related behavior. Given the cross-sectional design, these associations should not be interpreted causally; the findings nonetheless suggest that healthcare organizations may benefit from combining organizational-support practices with efforts that support proactive engagement among staff. As a primary goal, the abstract should render the general significance and conceptual advance of the work clearly accessible to a broad readership. References should not be cited in the abstract. Leave the Abstract empty if your article does not require one – please see the “Article types” on every Frontiers journal page for full details.

## 1 Introduction

Workplace safety is a persistent global public-health and economic concern. According to the International Labour Organization, approximately 2.93 million workers die annually from work-related factors, including occupational diseases and workplace accidents, while hundreds of millions experience non-fatal work-related injuries and illnesses (International Labour Organization, 2023). Healthcare workers are among the occupational groups exposed to substantial risks because their work involves regular contact with biological and chemical hazards, infectious materials, medical equipment, ergonomic demands, and psychosocial stressors (World Health Organization, 2021). Consequently, safety compliance behavior, defined as employees’ adherence to safety rules, procedures, and personal protective equipment requirements, represents a critical component of occupational safety in healthcare organizations (Neal and Griffin, 2006).

Despite the importance of safety compliance, the organizational and psychological factors associated with employees’ safety-related behavior remain insufficiently understood, particularly in developing country and non-Western contexts. Perceived organizational support (POS) refers to employees’ general belief that their organization values their contributions and cares about their well-being (Eisenberger et al., 1986; Rhoades and Eisenberger, 2002). Organizational Support Theory proposes that employees who perceive strong organizational support are more likely to reciprocate through positive attitudes and behaviors that contribute to organizational objectives. In healthcare settings, this reciprocity may be expressed through adherence to safety procedures, appropriate use of personal protective equipment, reporting of hazards, and participation in safety-improvement activities.

Recent empirical research supports the relevance of perceived organizational support to employee safety outcomes. For example, Adekanmbi et al. (2022) reported a significant relationship between perceived organizational support and the safety performance of healthcare workers. Their findings suggest that employees who perceive greater support from their organization may be more willing to engage in safety-oriented behavior. However, the strength of this relationship may be influenced by work-related stress, resource limitations, supervisory practices, and other contextual conditions. Thus, although organizational support may provide an important foundation for safety compliance, it may not fully explain why employees respond differently to similar organizational circumstances.

Individual characteristics may influence whether employees identify hazards, take initiative, and translate supportive organizational conditions into safety-related behavior. Proactive personality is an individual-difference characteristic that reflects a tendency to identify opportunities, take initiative, and attempt to influence or change the work environment (Bateman and Crant, 1993). In safety-sensitive environments, proactive employees may be more likely to identify hazards, report unsafe conditions, seek corrective solutions, and follow safety procedures even when direct supervision is limited. Empirical research has found that proactive personality is positively associated with safety compliance and safety participation (Bakidamteh et al., 2022). Moreover, research among nurses in Turkey indicated that proactive personality was related to safety behavior through safety attitudes, highlighting the relevance of this individual characteristic in healthcare safety contexts (Swieai et al., 2025). Most of this evidence has been generated in Western or East Asian contexts, with limited attention to healthcare workers in North African or Middle Eastern settings.

Nevertheless, the existing literature leaves several important questions unanswered. First, although previous studies have examined proactive personality as a predictor of safety-related outcomes, limited attention has been given to its potential role as a mediating mechanism between perceived organizational support and safety compliance. In other words, it remains unclear whether organizational support enhances safety compliance directly or whether supportive organizational conditions also encourage employees to adopt more proactive behavioral tendencies that subsequently promote safety compliance.

Second, empirical evidence concerning the relationships among perceived organizational support, proactive personality, and safety compliance remains limited in healthcare settings outside Western and East Asian contexts. This limitation is particularly evident in Libya and the broader North African region, where healthcare employees operate within distinctive institutional, organizational, and resource-related conditions. Findings obtained from other countries and occupational sectors may not be directly generalizable to employees working in Libyan public hospitals.

Third, previous findings do not provide a sufficiently consistent explanation of whether organizational support affects safety compliance primarily through a direct organizational pathway or through individual-level characteristics. This inconsistency indicates the need for an integrated model that examines organizational and individual predictors simultaneously. Addressing this issue may provide a more comprehensive understanding of how safety compliance develops in healthcare organizations.

Non-compliance with safety procedures in healthcare settings may expose employees and patients to preventable risks, including needlestick injuries, exposure to infectious materials, medication-related incidents, and other occupational hazards. Such incidents may also contribute to absenteeism, compensation costs, reduced work capacity, and disruption of healthcare services. Despite the importance of safety compliance in public hospitals, limited empirical research has examined the organizational and psychological factors associated with this behavior among healthcare workers in Libya.

In particular, it remains unclear whether employees’ perceptions of organizational support are associated with safety compliance directly or whether this association is partly explained by individual characteristics such as proactive personality. This lack of evidence creates a theoretical problem because relationships established in other countries and occupational contexts may not necessarily apply to Libyan healthcare organizations. It also creates a practical problem because hospital administrators and occupational-safety officers need context-specific evidence to determine whether safety improvement should focus primarily on organizational-support practices, employee participation, safety training, or a combination of these approaches. Therefore, the present study examines the relationships among perceived organizational support, proactive personality, and safety compliance behavior among healthcare employees in Libyan public hospitals.

The present study addresses three main gaps in the existing literature. First, although proactive personality has been examined as a predictor of safety-related outcomes, its potential mediating role in the relationship between perceived organizational support and safety compliance remains insufficiently investigated. Second, empirical evidence examining these variables simultaneously among healthcare workers in Libya and the broader North African context is scarce. Third, previous studies have not provided a consistent explanation of whether organizational support influences safety compliance through a direct organizational pathway, an individual-level pathway, or both. To address these gaps, the present study proposes and tests an integrated POS → PP → SC model. Specifically, it examines whether perceived organizational support is associated with safety compliance, whether perceived organizational support is related to proactive personality, whether proactive personality is associated with safety compliance, and whether proactive personality mediates the relationship between perceived organizational support and safety compliance. By testing these relationships within a single empirical model, the study provides context-specific evidence from Libyan public hospitals and contributes to a more comprehensive understanding of the organizational and individual factors underlying safety compliance in healthcare settings.

The study addresses the following research questions:

*RQ1*. Is perceived organizational support significantly associated with safety compliance among healthcare workers?

*RQ2*. Is perceived organizational support significantly associated with proactive personality among healthcare workers?

*RQ3*. Is proactive personality significantly associated with safety compliance among healthcare workers?

*RQ4*. Does proactive personality account for an indirect association between perceived organizational support and safety compliance?

The specific model is shown in Figure 1.

**Figure 1:**
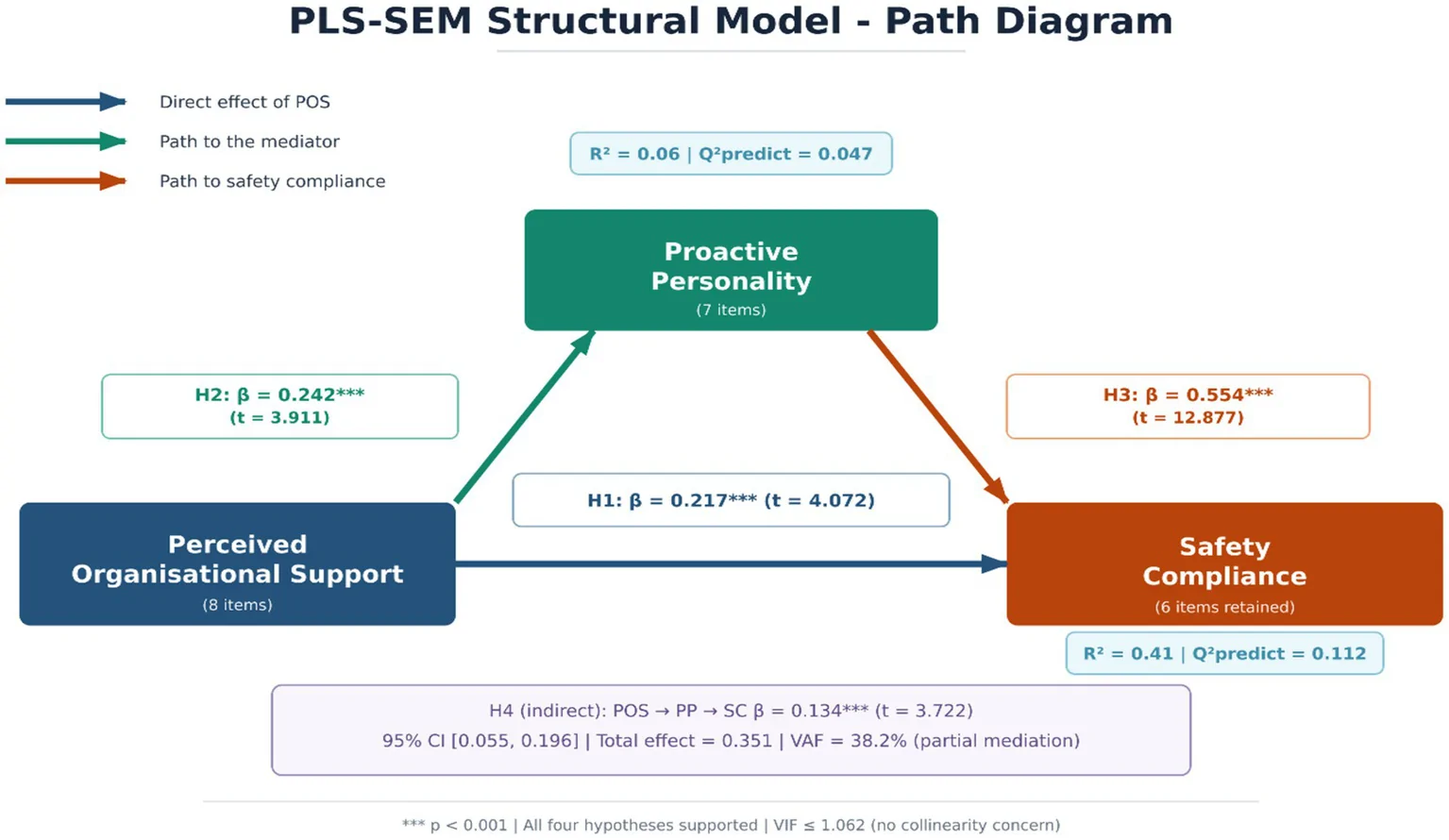
Model hypotheses.

### 1.1 Literature review

For Rather than reviewing the POS, proactive-personality, and safety-compliance literatures as separate, sequential strands, this section synthesizes them critically around the specific gap identified above. Kurtessis et al.’s (2017) meta-analysis of 558 studies established POS as a robust predictor of a wide range of employee attitudes and behaviors, grounded in social exchange theory: employees who perceive strong organizational support feel obliged to reciprocate through positive work behavior, including compliance with organizational rules. Applied to safety specifically, Qi et al. (2019) found a direct positive relationship between POS and safety performance across multiple industries, while Adekanmbi et al. (2022) reported a similar association among healthcare workers, moderated by pandemic-related stress. These studies converge on a direct-effect model but differ in the strength of the relationship reported, ranging from small to moderate, which suggests that unmeasured individual-level factors may account for some of this variability.

A second body of work has examined proactive personality as a predictor of safety-related outcomes largely independently of organizational-support research. Teye-Kwadjo and de Bruin (2021), studying fuel-station attendants, found that proactive personality was positively associated with both safety compliance and safety participation. Mo et al. (2023), in a multi-source study of construction workers, found that proactive personality was associated with safety behavior through safety self-efficacy and team-member exchange, and that this relationship was moderated by safety-specific transformational leadership. Notably, Mo et al. (2023) modeled proactive personality as a predictor operating through other mediators, rather than as a mediator of organizational support itself – the specification tested in the present study. Seibert et al. (1999) and Crant (1995) established proactive personality as a stable, generalizable individual-difference construct with consistent predictive validity for performance-related outcomes across occupational settings, supporting its relevance as an individual-level mechanism in the present model.

Taken together, this literature provides consistent evidence for a POS–safety link and, separately, for a proactive-personality–safety link, but very limited evidence on how these two predictors relate to one another in predicting safety compliance, and almost none from healthcare settings in North Africa.

### 1.2 Theoretical framework

This study is anchored primarily on Organizational Support Theory (OST; Eisenberger et al., 1986), which holds that employees form a global perception of how much their organization values their contribution and cares about their welfare, and that this perception obliges employees, through the norm of reciprocity, to direct positive effort – including safety-related effort – toward organizational goals. OST alone, however, does not fully explain why some employees translate perceived support into safety-compliant behavior more readily than others; individual differences plausibly condition this translation.

Trait Activation Theory (TAT; Tett and Burnett, 2003) is used here in a deliberately secondary and bounded role. TAT proposes that situational cues activate the behavioral expression of existing personality traits rather than altering the traits themselves. Proactive personality (PP) is conceptually distinguished here from proactive behavior: PP is treated as a relatively stable trait (Bateman and Crant, 1993), while its behavioral expression – including safety-relevant initiative – is understood as situationally activated (Fuller and Marler, 2009). Under this framing, a supportive organizational climate is proposed to activate the expression of proactive tendencies toward safety-relevant behavior, rather than to increase trait-level proactive personality itself. This distinction matters for how the mediation hypothesis (H4) below should be interpreted: it is proposed and tested as a statistical mediation model, consistent with much of the applied PLS-SEM literature, but conceptually it is more precisely understood as POS being associated with the behavioral activation of an existing trait. We return to this distinction, and to plausible alternative specifications (e.g., proactive personality as a moderator or as an antecedent measured concurrently with POS), in the Discussion and Limitations, since the cross-sectional design used here cannot adjudicate between these specifications.

### 1.3 Hypotheses development

As illustrated in Table 1, the literature provides strong evidence for the individual relationships between POS and employee outcomes (Kurtessis et al., 2017), between proactive personality and performance (Seibert et al., 1999), and between proactive personality and safety behavior (Teye-Kwadjo and de Bruin, 2021; Mo et al., 2023). Organizational Support Theory (Eisenberger et al., 1986) and Trait Activation Theory (Tett and Burnett, 2003) provide useful frameworks for understanding these relationships. However, an important gap remains. Although studies such as Mo et al. (2023) have examined proactive personality within structural models, they have not tested it as a mediator between perceived organizational support and safety compliance. In addition, the existing research has focused on Western or East Asian environments or on industries such as construction or oil and gas, with limited attention to healthcare environments in North Africa. This study addresses this gap by testing a model in which proactive personality acts as a psychological mechanism linking perceived organizational support and safety compliance among healthcare workers in Libya.

**Table 1:** Summarizes the studies most central to this synthesis.

No.	Study	Focus	Key findings	Theoretical contribution
1	Kurtessis et al. (2017)	Meta-analysis of POS	POS significantly influences various employee outcomes, including performance.	Comprehensive validation of organizational support theory.
2	Teye-Kwadjo and de Bruin (2021)	Proactive personality and safety	Proactive personality was positively associated with safety compliance and participation.	Application of trait activation theory to safety contexts.
3	Qi et al. (2019)	POS and safety performance	POS directly related to safety performance.	Extension of social exchange theory to safety domains.
4	Zohar (2010)	Safety climate	Defined safety climate and its relationship to safety outcomes.	Integration of climate theory with safety management.
5	Cropanzano and Mitchell (2005)	Social exchange theory	Comprehensive overview of social exchange theory in organizations.	Foundation for understanding reciprocal relationships in workplaces.
6	Tett and Burnett (2003)	Trait activation theory	Explained how situational cues activate the expression of personality traits.	Framework for understanding person–situation interactions.
7	Neal and Griffin (2006)	Safety climate and behavior	Safety climate positively associated with both safety compliance and participation over time.	Longitudinal integration of climate theory with safety research.
8	Griffin and Neal (2000)	Safety performance dimensions	Distinguished between safety compliance and safety participation.	Foundation for safety performance measurement.
9	Seibert et al. (1999), Crant (1995)	Proactive personality	Established proactive personality as a stable, generalizable construct.	Foundation for proactive-personality research in organizations.
10	Eisenberger et al. (1986)	Organizational support theory	Introduced the POS construct and organizational support theory.	Foundational work for organizational support research.
11	Mo et al. (2023)	Proactive personality and construction worker safety	PP associated with safety behavior; safety-specific transformational leadership moderates this association, while safety self-efficacy and team-member exchange mediate it.	Explains the dynamics of safety behavior by combining trait activation, social cognition, and social exchange.
12	Bateman and Crant (1993)	Proactive personality construct definition	Defined proactive personality as a stable disposition toward initiative-taking, distinct from situational proactive behavior.	Foundation for the trait/behavior distinction used in this study.

#### 1.3.1 Perceived organizational support and safety compliance

POS is defined as employees’ perceptions of the extent to which their organization values their contributions and cares about their well-being. Grounded in social exchange theory, POS is expected to encourage positive workplace behaviors such as commitment to safety and productivity (Kurtessis et al., 2017). Qi et al. (2019) and Adekanmbi et al. (2022) both report a positive direct association between POS and safety-related outcomes, although the latter found this relationship attenuated under high pandemic-related stress, suggesting the relationship is not uniformly strong across contexts. Employees in high-risk environments who perceive high organizational support are expected to be more likely to adhere to safety procedures, consistent with a sense of obligation to reciprocate the support they receive; the temporal ordering assumed here – that perceptions of support precede and relate to safety behavior, rather than the reverse – follows the theoretical logic of OST but cannot be confirmed with the cross-sectional data used in this study.

*H1*: Perceived organizational support is positively associated with safety compliance.

#### 1.3.2 Perceived organizational support and proactive personality

Although proactive personality is conceptualized as a stable trait, several studies report a positive association between POS and the behavioral expression of proactivity: employees who feel supported may be more willing to take initiative because they perceive lower risk in doing so (Ullah et al., 2020; Akgunduz et al., 2018). We therefore expect a positive cross-sectional association between POS and measured proactive personality in this sample, understood – per Section 1.2 – as reflecting the activation of proactive expression rather than a change in the underlying trait.

*H2*: Perceived organizational support is positively associated with proactive personality.

#### 1.3.3 Proactive personality and safety compliance

Proactive individuals are more likely to identify and address safety issues on their own initiative, independent of direct organizational prompting (Mo et al., 2023; Teye-Kwadjo and de Bruin, 2021). Within high-risk, tightly regulated environments such as healthcare, this tendency is expected to translate into stronger adherence to safety procedures.

*H3*: Proactive personality is positively associated with safety compliance.

#### 1.3.4 The indirect role of proactive personality

Building on H1–H3, and consistent with the bounded TAT framing in Section 1.2, we propose that part of the association between POS and safety compliance operates indirectly, through proactive personality. When employees perceive their organization as supportive, they may be more likely to express proactive tendencies, including initiative directed at safety-relevant behavior, which in turn is associated with safety compliance. Because the data are cross-sectional, this pathway is tested statistically as an indirect (mediated) association; it should not be interpreted as evidence of a causal sequence.

*H4*: Proactive personality mediates the association between perceived organizational support and safety compliance.

## 2 Study area and data collection

### 2.1 Study area

Ajdabiya is a major city in northeastern Libya, located about 150 km south of Benghazi. The city serves as the administrative center of al-Wahat District, with an estimated population of 140,000. It plays an important role in regional healthcare, as it houses several public hospitals, including Mohammed Al-Magarif Hospital, Al-Zweitina Hospital, and Al-Brega Hospital. These institutions employ a large number of healthcare workers, making Ajdabiya a suitable setting for studying occupational health and safety practices in the healthcare sector (Figure 2).

**Figure 2:**
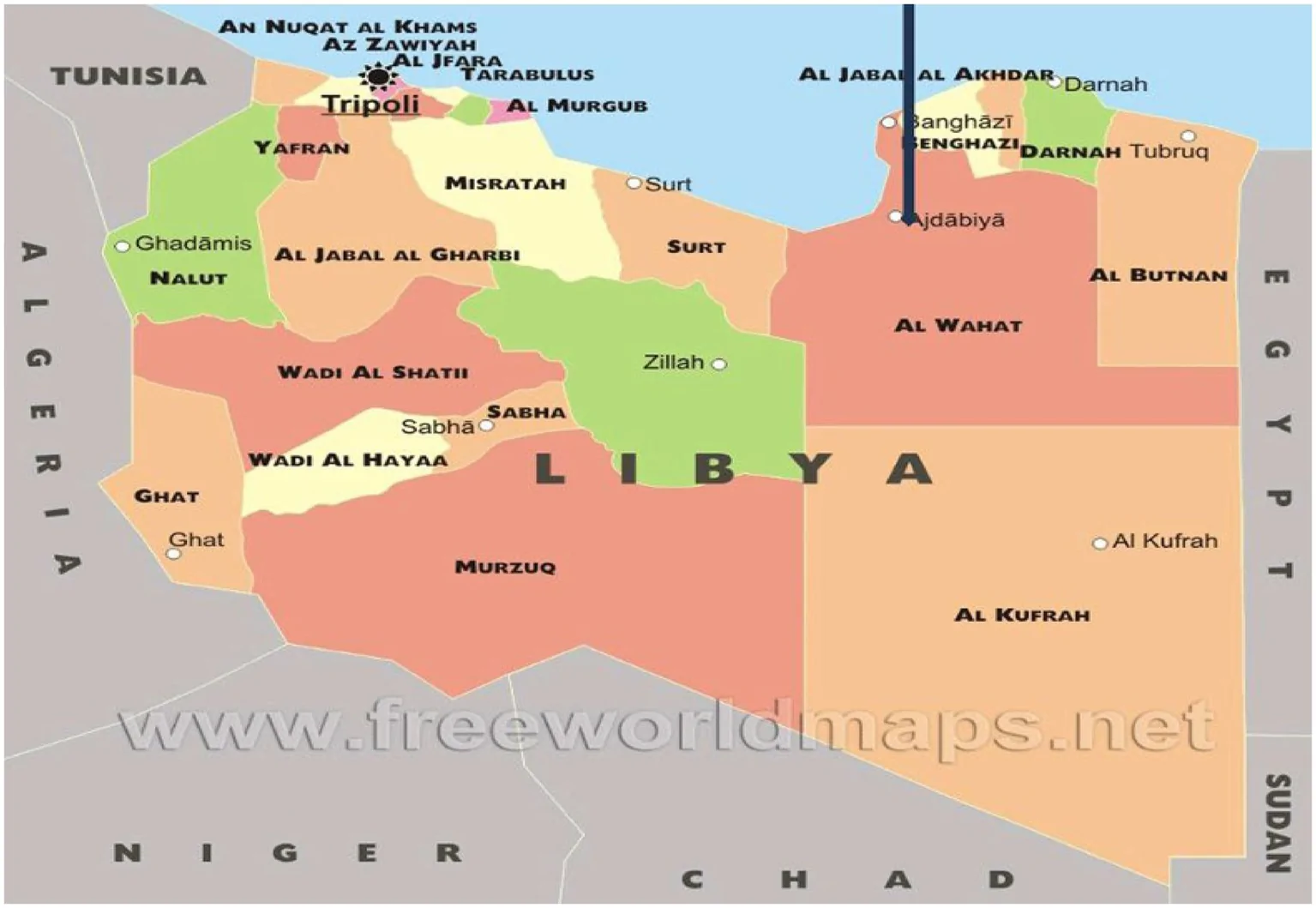
Study area (Google image 2026).

### 2.2 Study design and sampling

This was a hospital-based, cross-sectional survey study conducted among healthcare employees working in the three public hospitals described above.

### 2.3 Sample size

A simple random sampling technique was used to select participants from staff rosters obtained from the human resources departments of the three participating hospitals, which served as the sampling frame; every listed staff member had an equal probability of selection, and selection was not stratified by department or professional group. The total study population consisted of nearly 1,400 healthcare workers employed across the three hospitals. A total of 500 questionnaires were distributed, of which 309 were returned complete and valid for analysis, yielding a usable response rate of 61.8%. Questionnaires with substantial missing data were excluded before assembling the final analytic sample, and no further exclusions were required at the analysis stage. Any residual item-level missing data (isolated, unsystematic missing responses) were replaced using mean substitution, consistent with common PLS-SEM practice (Hair et al., 2019).

A response rate above 60% is generally considered acceptable in organizational survey research. According to Krejcie and Morgan’s (1970) table, a minimum sample of 302 respondents is sufficient to represent a population of approximately 1,400 at conventional confidence levels. We additionally computed a minimum sample-size requirement based on statistical power for the weakest structural path in the model (POS → Proactive Personality, observed *f*^2^ = 0.062, corresponding to *R*^2^ = 0.059), using the inverse-square-root method for PLS-SEM minimum sample size (Kock and Hadaya, 2018) and conventional power tables for a single-predictor regression *F*-test (*α* = 0.05, power = 0.80; Cohen, 1992); this indicates a minimum required sample of approximately 128–130 respondents. The achieved sample of 309 respondents therefore comfortably exceeds both the population-based and the power-based minimum requirements, and is also consistent with general PLS-SEM guidance that a sample above 200 supports stable and reliable path estimates.

### 2.4 Instruments and pilot study

A structured, self-administered questionnaire was used, comprising demographic items and measurement scales for the three study variables, all adapted from previously validated instruments and translated into Arabic and back-translated into English, followed by review by two bilingual subject-matter experts to confirm conceptual and linguistic equivalence for the Libyan healthcare context.

Prior to full administration, the instrument was piloted with 20 healthcare employees who were not included in the main analytic sample (Malhotra and Dash, 2011). Preliminary reliability for all three scales was acceptable (Cronbach’s *α* > 0.70), and minor wording clarifications were made to items that pilot respondents identified as ambiguous before the questionnaire was finalized for full administration (Nunnally and Bernstein, 1994).

### 2.5 Data collection procedure

Institutional approval to access staff and distribute the survey was obtained from the administration of each participating hospital, in addition to the ethical approvals described in Section 2.8 (Weijters and Baumgartner, 2012). Trained research assistants distributed paper-based questionnaires to randomly selected staff across day and night shifts to reduce shift-related sampling bias, accompanied by a cover letter explaining the study’s purpose, voluntary participation, and confidentiality. Completed questionnaires were collected in sealed boxes at each hospital over an eight-week data-collection period and were number-coded (with no personally identifying information retained) prior to data entry.

### 2.6 Measures and item structure

The demographic section comprised eight items: age, gender, marital status, educational level, healthcare profession, and years of experience, safety-training status, and employment contract type. The three main constructs (POS, PP, and SC) were measured using a total of 21 items (8, 7, and 6 items, respectively), as detailed in Table 2.

**Table 2:** Measurement instruments.

Construct	Source	Scale items	Anchors	Sample Item
Perceived Organizational Support (POS)	Eisenberger et al. (1986), Rhoades and Eisenberger (2002)	8	5-point Likert (1 = Strongly Disagree to 5 = Strongly Agree)	“My hospital really cares about my well-being.”
Proactive Personality (PP)	Bateman and Crant (1993), Crant (1995, short form)	7	5-point Likert (1 = Strongly Disagree to 5 = Strongly Agree)	“I am always looking for better ways to do things at work.”
Safety Compliance (SC)	Neal and Griffin (2006)	6	5-point Likert (1 = Strongly Disagree to 5 = Strongly Agree)	“I follow correct safety procedures while carrying out my job.” Prior to full administration, the instrument was piloted with 20 healthcare employees who were not included in the main analytic sample. Preliminary reliability for all three scales was acceptable (Cronbach’s α > 0.70), and minor wording clarifications were made to items that pilot respondents identified as ambiguous before the questionnaire was finalized for full administration.

### 2.7 Data analysis

Data were screened prior to substantive analysis. Questionnaires with extensive missing data were excluded before the final analytic sample was assembled (Section 2.3); residual item-level missingness was handled via mean substitution. Multivariate outliers were screened using standardized-score thresholds, and no cases were removed on this basis. Common method bias was assessed using the full collinearity VIF test (Kock, 2015): because all inner VIF values in the structural model were well below the conservative threshold of 3.3 (see Section 3.4), common method bias is unlikely to be a serious concern in this dataset (Podsakoff et al., 2003).

Descriptive and demographic statistics were computed in SPSS (Hair et al., 2018). All substantive measurement-model and structural-model analyses were conducted in SmartPLS [insert verified version] using partial least squares structural equation modeling (PLS-SEM), which is appropriate given the study’s prediction-oriented aim and the use of formative-adjacent, composite-based constructs. The measurement model was assessed first, examining indicator outer loadings (Hair et al., 2022), internal consistency reliability (Cronbach’s *α*, rho_A, and composite reliability), convergent validity (average variance extracted, AVE), and discriminant validity (the Fornell and Larcker, 1981 criterion and the heterotrait–monotrait, HTMT, ratio; Henseler et al., 2015; Dijkstra and Henseler, 2015). The structural model was then assessed for collinearity (inner VIF), path-coefficient significance, *R*^2^, effect sizes (*f*^2^), and out-of-sample predictive relevance (*Q*^2^predict, via PLSpredict) (Kline, 2016). The specific indirect effect of POS on SC through PP was tested using a bootstrapping procedure (Geisser, 1974; Shmueli et al., 2019; Stone, 1974) with 5,000 resamples (bias-corrected and accelerated, BCa, 95% confidence intervals), following current PLS-SEM mediation best practice (Hair et al., 2019; Nitzl et al., 2016) rather than the traditional Baron and Kenny (1986) approach.

### 2.8 Ethics statement

The study was approved by the Scientific Research Ethics Committee of Ajdabiya Health Service Administration (Reference No. 2024/983/3; date of approval: 8 July 2024), in accordance with the ethical standards of the University of Mediterranean Karpasia Institutional Review Board (IRB). All participants provided written informed consent prior to participation, and no personally identifiable data are reported in this manuscript.

## 3 Results

### 3.1 Demographic profile of respondents

To take Figure 3 summarizes the demographic characteristics of the 309 respondents. The sample was predominantly aged 26–35 years, female, and held a diploma-level qualification, consistent with the broader composition of the nursing-dominated healthcare workforce in Libya. No statistical tests of demographic subgroup differences were conducted; the descriptive profile is reported for sample-characterization purposes only.

**Figure 3:**
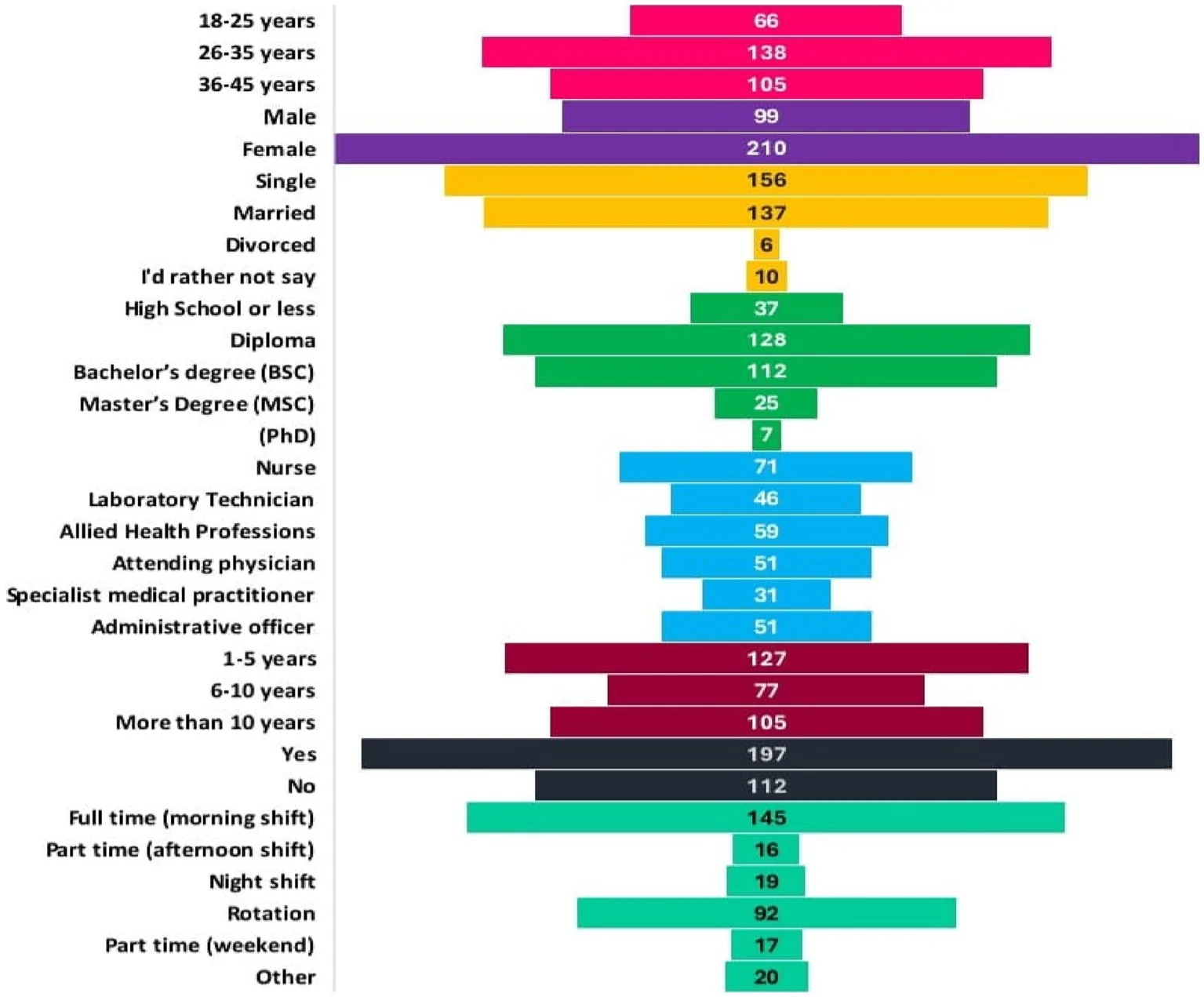
Combined demographic characteristics of respondents (*N* = 309). Percentages may not total exactly 100% because of rounding.

The largest share of respondents reported 1–5 years of experience (41.1%), followed by those with more than 10 years (34.0%) and those with 6–10 years (24.9%). The majority of respondents reported having received safety training (63.8%). Overall, the sample was professionally and educationally diverse, predominantly female and early-to-mid-career, and largely composed of staff who had received some form of prior safety training.

### 3.2 Measurement model assessment

Table 3 shows descriptive statistics for the three main constructs. All three constructs showed relatively high means (4.21–4.56 on a 5-point scale) and modest negative skew, indicating that most respondents rated themselves favorably on organizational support, proactivity, and safety compliance. Because all three measures are self-reported, either this pattern is consistent with genuinely high safety-supportive conditions or, alternatively, ceiling effects and/or social-desirability bias in responding to workplace-sensitive items; this issue is revisited in the Limitations (Section 4.5).

**Table 3:** Descriptive statistics for main constructs.

Construct	Mean	Std. Deviation	Skewness	Kurtosis
Perceived Organizational Support (POS)	4.32	1.25	−0.42	0.18
Proactive Personality (PP)	4.56	1.18	−0.35	0.21
Safety Compliance (SC)	4.21	1.32	−0.51	0.32

The measurement model initially included 21 (8 for POS, 7 for PP, and 6 for SC) original indicators were retained in the final measurement model; none were removed. Table 4 reports the standardized outer loadings, with bootstrapped *t*- and *p*-values, for each indicator (Table 5).

**Table 4:** Standardized outer loadings.

Item	Construct	Loading	*t*-value	*p*-value
POS1	POS	0.620	8.832	<0.001
POS2	POS	0.587	7.122	<0.001
POS3	POS	0.697	13.902	<0.001
POS4	POS	0.519	7.752	<0.001
POS5	POS	0.471	5.728	<0.001
POS6	POS	0.794	20.181	<0.001
POS7	POS	0.865	45.239	<0.001
POS8	POS	0.836	28.642	<0.001
PP1	PP	0.709	12.124	<0.001
PP2	PP	0.688	11.112	<0.001
PP3	PP	0.699	12.621	<0.001
PP4	PP	0.700	12.131	<0.001
PP5	PP	0.733	17.838	<0.001
PP6	PP	0.733	20.919	<0.001
PP7	PP	0.779	26.867	<0.001
SC1	SC	0.699	20.512	<0.001
SC2	SC	0.533	7.990	<0.001
SC3	SC	0.589	10.259	<0.001
SC4	SC	0.839	37.858	<0.001
SC5	SC	0.862	43.447	<0.001
SC6	SC	0.856	40.053	<0.001

**Table 5:** Reliability and convergent validity.

Construct	Items	Cronbach’s *α*	rho_A	CR	AVE
POS	8	0.836	0.860	0.873	0.473
PP	7	0.853	0.870	0.883	0.519
SC	6	0.833	0.866	0.876	0.550

α, rho_A, and CR > 0.70 for all constructs; AVE > 0.50 for PP and SC, marginally below 0.50 for POS (see text).

Figure 4 presents the estimated measurement model, showing each retained indicator alongside its standardized loading on Perceived Organizational Support, Proactive Personality, and Safety Compliance. The model shows that all retained indicators load exclusively on their respective constructs, with no evidence of cross-loadings. Following the removal of SC7, six indicators rather than seven represent Safety Compliance.

**Figure 4:**
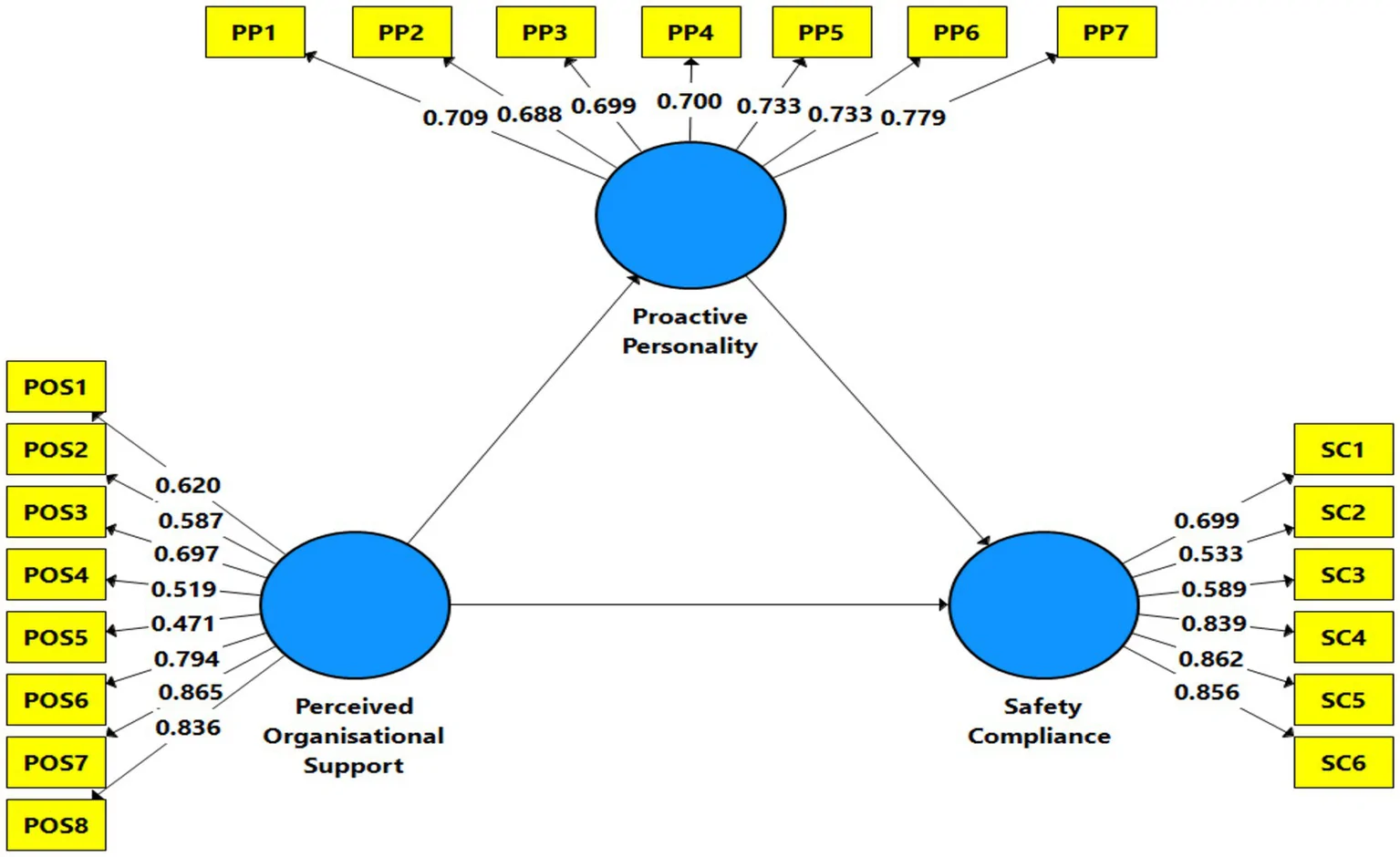
Measurement model.

### 3.3 Discriminant validity

Discriminant validity was evaluated using both the Fornell–Larcker criterion and the HTMT ratio (Henseler et al., 2015). The square root of AVE for each construct (Table 6, diagonal) exceeded its correlations with the other constructs, supporting discriminant validity. All HTMT ratios (Table 7) were below the conservative 0.85 threshold, confirming that the constructs are empirically distinct.

**Table 6:** Discriminant validity – Fornell–Larcker criterion.

Construct	POS	PP	SC
POS	0.688		
PP	0.242	0.721	
SC	0.351	0.606	0.741

Diagonal values are the square root of AVE.

**Table 7:** Heterotrait–Monotrait (HTMT) Ratio.

Construct pair	HTMT
Proactive Personality ↔ POS	0.282
Safety Compliance ↔ POS	0.410
Safety Compliance ↔ Proactive Personality	0.616

All HTMT values are below the 0.85 threshold, confirming discriminant validity.

### 3.4 Structural model, collinearity, and hypothesis testing

Inner VIF values, reported by predictor and outcome, are shown in Table 8; all values (1.000–1.062) were well below both the conservative structural threshold of 3 and the stricter 3.3 threshold used as a common-method-bias check (Kock, 2015), indicating that multicollinearity and common method bias are unlikely to be materially distorting the path estimates.

**Table 8:** Inner variance inflation factors (VIF).

Predictor	→ Proactive Personality	→ Safety Compliance
Perceived Organizational Support	1.000	1.062
Proactive Personality	–	1.062

Table 9 reveals that perceived organizational support (POS) positively impacts proactive personality (PP) and safety compliance (SC). PP strongly influences SC, surpassing the direct POS to SC effect. Thus, all three hypotheses (H1, H2, and H3) receive support, indicating significant relationships among the variables.

**Table 9:** Hypothesis testing (structural model).

Hypothesis/path	*β*	*t*-value	*p*-value	95% CI (BCa)	Result
H1: POS → SC	0.217	3.941	<0.001	[0.098, 0.315]	Supported
H2: POS → PP	0.242	3.922	<0.001	[0.091, 0.345]	Supported
H3: PP → SC	0.554	13.130	<0.001	[0.457, 0.629]	Supported

### 3.5 Structural model, collinearity, and hypothesis testing

Table 10 shows that the model explains 5.9% of the variance in proactive personality (PP; *R*^2^adj = 0.056) and 41.2% of the variance in safety compliance (SC; *R*^2^adj = 0.408) (Table 11). Table 12 reports effect sizes (*f*^2^) for each individual path rather than a single aggregate value per outcome: the effect of proactive personality on safety compliance was large (*f*^2^ = 0.490), while the effects of POS on proactive personality (*f*^2^ = 0.062) and on safety compliance (*f*^2^ = 0.075) were both small (Cohen, 1988). Out-of-sample predictive relevance (*Q*^2^predict, via PLSpredict) was positive for both.

**Table 10:** Model explanatory and predictive power.

Endogenous variable	*R* ^2^	*R*^2^ Adjusted	*Q*^2^predict
Proactive Personality (PP)	0.059	0.056	0.046
Safety Compliance (SC)	0.412	0.408	0.110

**Table 11:** Effect sizes (*f*^2^) by path.

Path	*f* ^2^	Interpretation (Cohen, 1988)
POS → PP	0.062	Small
POS → SC	0.075	Small
PP → SC	0.490	Large

**Table 12:** Effect sizes (*f*^2^) by Path.

Path	*f* ^2^	Interpretation (Cohen, 1988)
POS → PP	0.062	Small
POS → SC	0.075	Small
PP → SC	0.490	Large

Table 10 indicates the model accounts for 5.9% variance in proactive personality and 41.2% in safety compliance.

The effect of proactive personality on safety compliance is large (*f*^2^ = 0.490), while POS effects are small. Out-of-sample predictive relevance is positive for both variables, indicating model robustness.

### 3.6 Explanatory and predictive power

Table 13 shows that the model explains 5.9% of the variance in proactive personality (PP; *R*^2^adj = 0.056) and 41.2% of the variance in safety compliance (SC; *R*^2^adj = 0.408). Table 12 reports effect sizes (*f*^2^) for each individual path rather than a single aggregate value per outcome (Falk and Miller, 1992): the effect of proactive personality on safety compliance was large (*f*^2^ = 0.490), while the effects of POS on proactive personality (*f*^2^ = 0.062) and on safety compliance (*f*^2^ = 0.075) were both small (Cohen, 1988). Out-of-sample predictive relevance (*Q*^2^predict, via PLSpredict) was positive for both (Geisser, 1974).

**Table 13:** Model explanatory and predictive power.

Endogenous variable	*R* ^2^	*R*^2^ adjusted	*Q*^2^predict
Proactive Personality (PP)	0.059	0.056	0.046
Safety Compliance (SC)	0.412	0.408	0.110

### 3.7 Mediation analysis

To test H4, a bootstrapping procedure with 5,000 resamples (BCa 95% confidence intervals) was used to test the indirect association between Perceived Organizational Support (POS) and Safety Compliance (SC) through Proactive Personality (PP). As shown in Table 14, the path (POS → PP) was 0.242 [0.091, 0.345], the b path (PP → SC) was 0.554 [0.457, 0.629], and the resulting indirect effect (a × b) was 0.134, *t* = 3.711, *p* < 0.001, 95% CI [0.051, 0.196], which does not contain zero and is therefore statistically significant. The direct effect of POS on SC remained significant after accounting for the mediator (*β* = 0.217, *p* < 0.001), and the total effect of POS on SC was 0.351 (direct + indirect). Because both the direct and indirect effects are significant and share the same sign, this pattern is classified as complementary (partial) mediation (Hair et al., 2019; Nitzl et al., 2016), rather than assessed using the older Zhao et al. (2010) and Baron and Kenny (1986) causal-steps approach. The indirect effect accounted for approximately 38.2% of the total effect (variance accounted for, VAF = 0.134/0.351).

**Table 14:** Mediation analysis (H4: POS → PP → SC).

Path	*β*	*t*-value	*p*-value	95% CI (BCa)	Result
a path: POS → PP	0.242	3.922	<0.001	[0.091, 0.345]	Significant
b path: PP → SC	0.554	13.130	<0.001	[0.457, 0.629]	Significant
Direct effect: POS → SC	0.217	3.941	<0.001	[0.098, 0.315]	Significant
Indirect effect: POS → PP → SC	0.134	3.711	<0.001	[0.051, 0.196]	Significant
Total effect: POS → SC	0.351	–	–	–	–

Given the cross-sectional design, this pattern is reported as a statistically significant indirect association consistent with partial mediation, rather than as evidence of causal mediation; the data cannot establish the temporal precedence that a causal mediation claim would require.

Given the cross-sectional design, this pattern is reported as a statistically significant indirect association consistent with partial mediation, rather than as evidence of causal mediation; the data cannot establish the temporal precedence that a causal mediation claim would require.

## 4 Discussion

This study examined the associations among perceived organizational support (POS), proactive personality (PP), and safety compliance (SC) among healthcare workers in Libyan public hospitals. POS was positively associated with both PP and SC, while PP showed a positive association with SC. The indirect association between POS and SC through PP was also statistically significant. Because the study used a cross-sectional design, these findings are interpreted as statistical associations rather than evidence of causal relationships or temporal mediation.

### 4.1 Direct associations of POS and PP with SC

The POS was positively associated with SC ([\beta = 0.217], [*p* < 0.001]), supporting H1 and aligning with previous research linking organizational support with safety-related outcomes (Qi et al., 2019; Adekanmbi et al., 2022). The associated effect size was small ([*f*^2^ = 0.075]), suggesting that POS made a statistically significant but relatively limited contribution to SC after PP was included in the model. This finding is consistent with the broader organizational-support literature, which indicates that POS can be associated with desirable employee behaviors, although the magnitude of these associations may vary according to occupational and organizational context (Kurtessis et al., 2017).

PP showed a considerably stronger statistical association with SC ([\beta = 0.554], [*f*^2^ = 0.490]), supporting H3. Among the predictors included in the model, this was the strongest path. One possible interpretation, consistent with the bounded application of Trait Activation Theory, is that employees who are more inclined to identify opportunities, take initiative, and address problems may be more likely to express safety-relevant behaviors in a highly regulated healthcare environment. However, because PP and SC were measured concurrently, the result should not be interpreted as evidence that proactive personality causes safety compliance. It indicates only that the two constructs were strongly associated in this sample.

### 4.2 Indirect association through PP

The indirect association between POS and SC through PP was statistically significant ([\beta = 0.134], [*p* < 0.001]), accounting for approximately 38.2% of the total association. The significant positive direct and indirect effects, together with their common direction, are consistent with complementary partial mediation. This result suggests that the association between organizational support and safety compliance may be partly related to employees’ proactive orientation or its expression in the workplace.

Nevertheless, the mediation result should be interpreted cautiously. The cross-sectional design does not establish whether POS preceded PP or SC, and it cannot determine whether POS influences the development of proactive personality, whether proactive employees perceive greater organizational support, or whether a third variable contributes to all three constructs. Therefore, the result is best described as a statistically significant indirect association rather than a confirmed causal mechanism.

### 4.3 Theoretical implications

The findings contribute to Organizational Support Theory by extending its application to safety compliance among healthcare workers in an under-researched Libyan public-hospital context. The positive association between POS and SC is consistent with the proposition that employees who perceive their organization as valuing their contributions and caring about their well-being may be more willing to engage in behaviors that support organizational functioning, including adherence to safety procedures. However, the relatively small effect size indicates that organizational support is unlikely to be the only relevant factor associated with safety compliance.

The findings also provide a bounded application of Trait Activation Theory. Rather than suggesting that organizational support changes a stable personality trait, the results are consistent with the possibility that supportive work conditions are associated with the expression of proactive tendencies in safety-relevant situations. This interpretation is theoretically more cautious because proactive personality is generally conceptualized as a relatively stable individual difference, whereas proactive behavior can vary according to situational cues.

The statistical model also leaves room for alternative theoretical specifications. For example, PP could be examined as a moderator of the POS–SC association, or it could be modeled as a concurrent antecedent of SC alongside POS. The present cross-sectional data cannot distinguish confidently between these alternatives. Future longitudinal and multi-wave studies should therefore examine temporal ordering and compare mediation, moderation, and competing antecedent models.

### 4.4 Practical implications

The findings have practical implications for hospital administrators, department managers, supervisors, occupational-safety officers, and staff-development units. First, the positive association between POS and SC suggests that safety management should be supported by visible organizational and supervisory practices. Administrators can strengthen this support by ensuring timely access to personal protective equipment, responding promptly to reported safety concerns, recognizing employees’ safety-related contributions, and demonstrating concern for staff well-being. These actions are directly relevant to the POS–SC association observed in the study, although their causal effectiveness requires evaluation in future intervention research.

Second, the significant association between POS and PP indicates the value of creating formal opportunities for employee initiative. Hospitals could establish safety committees that include representatives from different professional groups, introduce structured channels for reporting hazards and near misses, and require documented feedback on the concerns raised by staff. Such mechanisms would allow employees to participate in identifying problems and proposing improvements, thereby providing practical opportunities for proactive engagement rather than relying only on top-down instructions.

Third, the relatively strong statistical association between PP and SC suggests that safety programs should encourage employees to identify hazards, anticipate potential failures, and take appropriate preventive action. Training units and safety officers could incorporate scenario-based exercises, case discussions, and simulations focused on hazard recognition and corrective action. These activities should complement, rather than replace, adequate staffing, equipment, supervision, and formal safety procedures. Recruitment and promotion decisions should also avoid treating proactive personality as a substitute for organizational responsibility or as a basis for excluding employees.

Finally, the significant indirect association suggests that a combined approach may be more appropriate than relying on a single safety intervention. Hospital leaders can link supportive supervision with non-punitive reporting systems, regular safety-feedback meetings, and employee participation in reviewing procedures. The primary users of these findings are hospital administrators and occupational-safety personnel, who can use them to design support and participation mechanisms tailored to local hospital conditions. Because this study was cross-sectional, these recommendations should be regarded as evidence-informed applications of the observed associations, not as interventions whose effectiveness has already been established.

### 4.5 Limitations

Several limitations should be considered when interpreting the findings. First, the cross-sectional design involved data collection at a single point in time. Consequently, the study cannot establish causal relationships or temporal precedence among POS, PP, and SC. This limitation is particularly important for the mediation hypothesis because the statistical model does not demonstrate that POS preceded PP or that PP preceded SC. Longitudinal, multi-wave, or experimental designs are needed to evaluate the proposed ordering more rigorously.

Second, all variables were measured using self-report questionnaires administered through the same instrument. Although the first factor in Harman’s test accounted for 28.4% of the variance and the maximum full-collinearity VIF was 1.062, these diagnostics do not completely eliminate the possibility of common method variance. Future studies should combine employee surveys with supervisor assessments, safety records, observation-based measures, incident reports, or compliance audits. The descriptive results also showed different response patterns across the constructs, with relatively low POS, relatively high PP, and moderate SC scores. These patterns may reflect actual differences in the constructs, but ceiling effects and social-desirability bias cannot be ruled out, particularly for self-reported safety behavior.

Third, the study was conducted in three public hospitals in Ajdabiya, Libya. The findings may therefore not generalize to private hospitals, other Libyan regions, or healthcare systems with different organizational and resource conditions. In addition, although the usable response rate was approximately 62%, respondents and non-respondents were not compared; therefore, non-response bias cannot be excluded.

Fourth, the measurement scales were translated into Arabic, back-translated, and reviewed by bilingual experts. However, formal cross-cultural measurement-invariance testing was not conducted. Future research should assess measurement equivalence and further validate the instruments among different professional groups and healthcare settings in Libya.

Finally, safety-training status was measured only as a received/not-received category and was used for descriptive purposes. Information about training content, duration, quality, and recency was not collected. As a result, the study could not determine whether the nature or quality of safety training influenced POS, PP, or SC. Moreover, the mediation specification involving PP was only one theoretically plausible model; moderation and alternative antecedent models should be examined in future research.

## 5 Conclusion

This study found positive associations among perceived organizational support, proactive personality, and safety compliance among healthcare workers in Libyan public hospitals. POS was associated with SC both directly and indirectly through PP, while PP showed the strongest statistical association with SC among the paths examined. The indirect effect was consistent with complementary partial mediation; however, the cross-sectional design does not permit causal or temporal conclusions.

The findings suggest that hospital safety management may benefit from combining organizational-support practices with opportunities for proactive employee participation, hazard reporting, and safety improvement. These implications should be interpreted as evidence-informed recommendations rather than confirmed intervention effects. Future longitudinal, multi-source, and multi-site research should test the temporal ordering of the variables, examine alternative mediation and moderation models, and determine whether targeted organizational interventions are associated with measurable improvements in safety compliance.

## Data Availability

The original contributions presented in the study are included in the article/supplementary material, further inquiries can be directed to the corresponding author.
